# Mammary Glands of Women, Female Dogs and Female Rats: Similarities and Differences to Be Considered in Breast Cancer Research

**DOI:** 10.3390/vetsci10060379

**Published:** 2023-05-30

**Authors:** Tiago Ferreira, Adelina Gama, Fernanda Seixas, Ana I. Faustino-Rocha, Carlos Lopes, Vítor M. Gaspar, João F. Mano, Rui Medeiros, Paula A. Oliveira

**Affiliations:** 1 Centre for the Research and Technology of Agro-Environmental and Biological Sciences (CITAB), University of Trás-os-Montes and Alto Douro (UTAD), 5000-801 Vila Real, Portugal; 2 Institute for Innovation, Capacity Building and Sustainability of Agri-Food Production (Inov4Agro), University of Trás-os-Montes and Alto Douro (UTAD), 5000-801 Vila Real, Portugal; 3 Molecular Oncology and Viral Pathology Group, Research Center of IPO Porto (CI-IPOP)/RISE@CI IPOP (Health Research Network), Portuguese Oncology Institute of Porto (IPO Porto), Porto Comprehensive Cancer Center (Porto.CCC), 4200-072 Porto, Portugal; 4 Department of Chemistry, CICECO—Aveiro Institute of Materials, University of Aveiro, Campus Universitário de Santiago, 3810-193 Aveiro, Portugal; 5 Animal and Veterinary Research Centre (CECAV), University of Trás-os-Montes and Alto Douro (UTAD), 5000-801 Vila Real, Portugal; 6 Associate Laboratory for Animal and Veterinary Sciences (AL4AnimalS), University of Trás-os-Montes and Alto Douro (UTAD), 5000-801 Vila Real, Portugal; 7 Department of Zootechnics, School of Sciences and Technology, University of Évora, 7004-516 Évora, Portugal; 8 Comprehensive Health Research Center, 7004-516 Évora, Portugal; 9 Portuguese Oncology Institute of Porto (IPO Porto), 4200-072 Porto, Portugal; 10 Faculty of Medicine, University of Porto (FMUP), 4200-319 Porto, Portugal; 11 Research Department of the Portuguese League against Cancer—Regional Nucleus of the North (Liga Portuguesa Contra o Cancro—Núcleo Regional do Norte), 4200-177 Porto, Portugal; 12 Virology Service, Portuguese Institute of Oncology (IPO), 4200-072 Porto, Portugal; 13 Biomedical Research Center (CEBIMED), Faculty of Health Sciences of the Fernando Pessoa University, 4249-004 Porto, Portugal

**Keywords:** breast, breast cancer, female dog, female rat, mammary gland, woman

## Abstract

**Simple Summary:**

Breast cancer research is frequently performed using a variety of models, including animal models, in an attempt to provide information that can be translated into human clinical practice. This review aims to demonstrate the similarities and differences in the anatomy of the mammary glands of women, female dogs and female rats, as well as the epidemiology, risk factors, and histopathological features of breast/mammary cancer in each model. It also demonstrates the advantages and disadvantages of each model, as they can serve as sources for several in vitro models, further increasing the translational potential of these models.

**Abstract:**

Breast cancer is one of the most common and well-known types of cancer among women worldwide and is the most frequent neoplasm in intact female dogs. Female dogs are considered attractive models or studying spontaneous breast cancer, whereas female rats are currently the most widely used animal models for breast cancer research in the laboratory context. Both female dogs and female rats have contributed to the advancement of scientific knowledge in this field, and, in a “One Health” approach, they have allowed broad understanding of specific biopathological pathways, influence of environmental factors and screening/discovery of candidate therapies. This review aims to clearly showcase the similarities and differences among woman, female dog and female rat concerning to anatomical, physiological and histological features of the mammary gland and breast/mammary cancer epidemiology, in order to better portray breast tumorigenesis, and to ensure appropriate conclusions and extrapolation of results among species. We also discuss the major aspects that stand out in these species. The mammary glands of female dogs and women share structural similarities, especially with respect to the lactiferous ducts and lymphatic drainage. In contrast, female rats have only one lactiferous duct per nipple. A comprehensive comparison between humans and dogs is given a special focus, as these species share several aspects in terms of breast/mammary cancer epidemiology, such as age of onset, hormonal etiology, risk factors, and the clinical course of the disease. Holistically, it is clear that each species has advantages and limitations that researchers must consider during the development of experimental designs and data analysis.

## 1. Introduction

Breast/mammary cancer is the most frequently diagnosed cancer in women and in female dogs [1,2]. Due to the clinical importance of human breast cancer, mammary glands have been studied extensively. Since experimental studies with women are not allowed for ethical reasons, female laboratory animals play a key role in this research area, because they can be sacrificed at particular time points, providing samples to develop alternative in vitro models. Female dogs are considered good spontaneous models for studying mammary cancer, and can be used to evaluate new therapies [3]. Rats and mice develop mammary cancer when exposed to specific chemical carcinogens and have several advantages when compared to dogs; specifically, high reproducibility, specificity and ease of handling [4,5]. To ascertain both the validity and the limitations of spontaneous or induced animal models for studying breast cancer in women, it is essential to be aware of the similarities and differences in the mammary glands among female rats, female dogs and women. Although there are cases of breast cancer in men and male dogs, its incidence is much lower. In fact, breast cancer in men occurs at a rate of 1 in 100 female cases (corresponding to 1% of all breast cancers). Despite the reduced incidence, men have worse survival outcomes than women [6,7]. In male dogs, mammary tumors are also rare, but they are generally benign and have better survival outcomes [8,9,10]. Female dogs are approximately 62 times more susceptible than male dogs to developing mammary tumors [8]. As in men and male dogs, spontaneous mammary tumors in male rats are uncommon. This sex difference in the incidence of breast/mammary tumors in men, male dogs and male rats depends on their hormonal environment, including sexual dimorphism of the rat mammary gland [11].

Taking this into account, in this review, an overview will be provided of the anatomical and physiological characteristics of the female mammary gland of these three species. Before addressing the main topic of this review, it is worth noting that terminology is an important point to be considered when comparing breast cancer between humans and animals. In the case of animals, the most correct terminology is the mammary gland, but for humans, the term breast should be used. Despite this, such terminology is applied haphazardly and without scientific foundation in many published works.

## 2. Breast/Mammary Gland Anatomy

**Figure 1:**
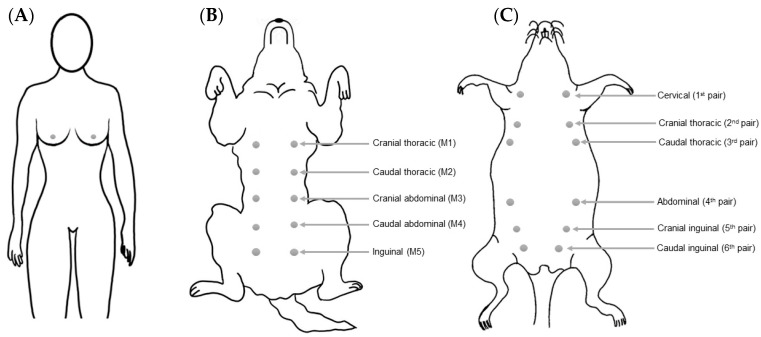
Schematic representation of the anatomical distribution and denomination of the mammary glands in women (**A**), female dogs (**B**) and female rats (**C**).

Mammary glands are found exclusively in mammals (class *Mammalia*), and constitute the glandular tissue of the mama (from the Latin—mamma) [12,13]. The human breast is homologous to the canine/rodent mamma, being composed of a mammary gland, connective tissue, skin and a nipple. Women (*Homo sapiens*) have two breasts, lateral to the median sagittal plane and located in the anterior thoracic wall (Figure 1A). They are supported by the pectoralis major muscle, the largest muscle of the anterior chest wall, which extends from the second to the sixth rib. Dogs (*Canis lupus familiaris*) have two mammary chains, left and right, with five mammae in each one, with a total of two thoracic pairs (M1 and M2), two abdominal pairs (M3 and M4) and one inguinal pair (M5) (Figure 1B). However, four and six pairs of glands have previously been described in some dogs, without any association with the breed [14]. With respect to their size, the inguinal pairs are larger than the abdominal pairs, and the thoracic pairs are the smallest. In non-lactating females, the position of the mammae can be identified by the nipples [15].

Female rats (*Rattus norvegicus*) have six pairs of bilaterally symmetrical mammae (left and right), located along the ventral body wall, which extend from the thoracic to the inguinal region (Figure 1C) [16]. A greater amount of gland tissue is present in the abdominal–inguinal region when compared with the thoracic region [17]. Unlike in women, the mammary glands of female rats are poorly developed, and can only be detected by the presence of nipples [16,18]. There are two nomenclatures used to describe the female rats’ mammae: (1) cervical, cranial thoracic, caudal thoracic, abdominal, cranial inguinal and caudal inguinal glands; and (2) numbering of the corresponding nipples from anterior to posterior as left first, right first, left second, right second, and so on until the sixth gland [18,19]. The mammary glands may extend laterally to the sides of the body, especially in lactating female rats [20]. In mice (Mus musculus), the anatomical location and distribution of the mammae are similar to that described for rats, with the exception, however, of a difference in the total number of mammary glands, as mice have five pairs (one cervical, two thoracic, and two abdominal–inguinal) instead of the six pairs described for rats [21].

## 3. Physiological and Histological Features of the Mammary Gland

**Figure 2:**
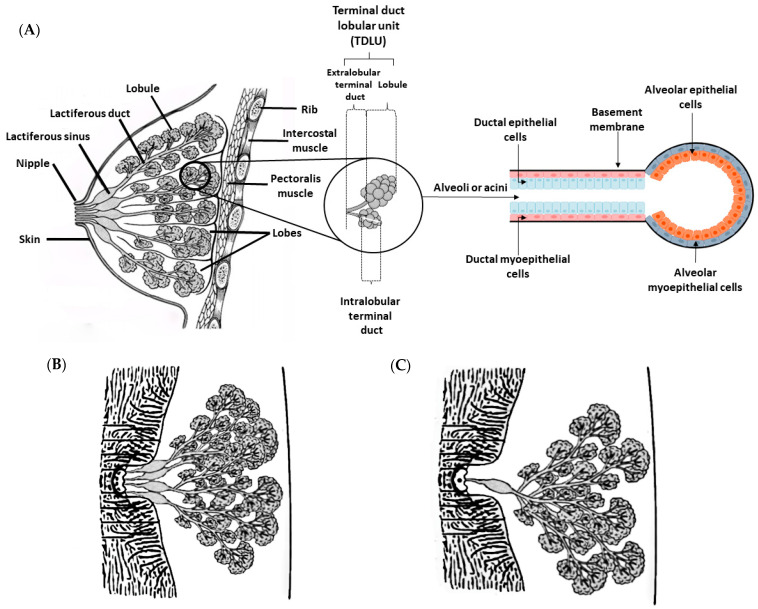
Schematic representation of the adult mammary gland. (**A**) Hierarchical organization of the mammary gland in an adult woman. The mammary gland is located in the anterior thoracic wall, superficial to the pectoralis muscles. The alveoli or acini, which are composed of a single layer of alveolar epithelial cells surrounded by myopepithelial cells and the basement membrane, are the basic components of the mature mammary gland. Myoepithelial cell contractions release milk to the ducts, and then to the nipple, while the basement membrane maintains cell contact with the extracellular environment. The morphofunctional unit—the terminal duct lobular unit—consists of a lobule associated with intralobular and extralobular terminals, and each lobe contains a lactiferous duct that drains into the nipple through the lactiferous sinus. (**B**) The mammary glands of female dogs have fewer lactiferous ducts than the mammary glands of women. (**C**) The mammary glands of female rats only have one lactiferous duct *per* nipple.

**Figure 3:**
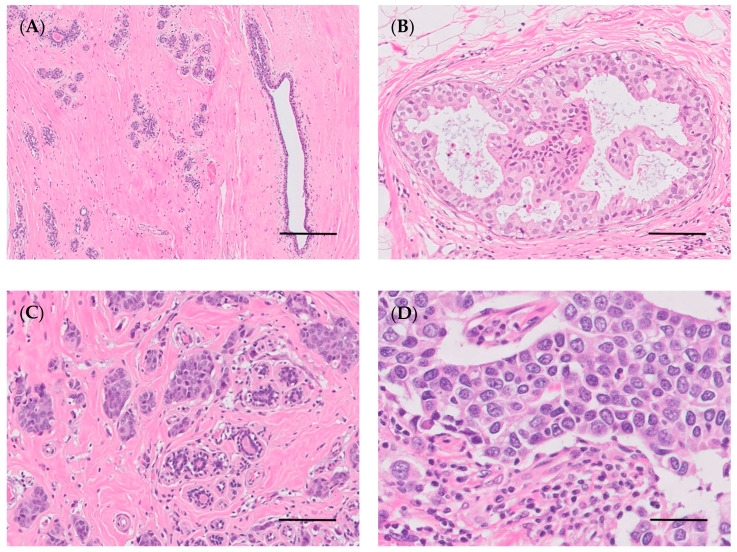
Woman’s breast. (**A**) Normal breast (bar = 200 µm); (**B**) ductal carcinoma in situ, micropapillary (bar = 100 µm); (**C**) invasive carcinoma of no special type, IC-NST (bar = 100 µm); (**D**) high-grade carcinoma (bar = 50 µm). Hematoxylin and eosin staining.

The mammary gland is a modified sweat gland that is exclusive of mammals. This gland secretes milk to nourish offspring and provide immune support [22]. The mammary gland is the target of different hormones, such as prolactin, estrogen and progesterone, which control its development and action [23]. Therefore, the mammary glands are hormone-dependent, and their development and growth are strongly influenced by the estrous cycle and pregnancy [24]. At birth, the mammary glands are rudimentary and nearly identical between women and men [25]. In women, breast development begins at puberty, when the mammary gland is exposed to estrogen and progesterone, but its terminal differentiation only occurs during pregnancy [26]. As mentioned above, the mammary glands of men remain rudimentary throughout their entire life [27]. Histologically, the breast in women is consists of three main components: skin, glandular tissue and supportive connective tissue [28,29]. The skin presents sebaceous and sweat glands. The glandular tissue consists of branching ducts and terminal secretory lobules, and the supportive connective tissue is responsible for the shape, size and support of the breast. Adipocytes, fibroblasts, endothelial cells, innate immune cells (including macrophages and mast cells), and peripheral nerves are the main components of connective tissue [30,31]. The human mammary gland is composed of tubuloalveolar glands and consists of a network of branched ducts from alveoli that extend through smaller ducts to the nipple [29]. The structures called alveoli, or acini, produce milk during lactation, through the action of the hormone prolactin [32]. They are formed by an inner layer—of luminal cells—and an outer layer—of myoepithelial cells—which is surrounded by the basement membrane, which separates the epithelium from the extracellular matrix [33]. The inner layer has a simple cubic or columnar secretory epithelium and it is responsible for the production of milk, while the myoepithelial cell layer (spindle-shaped cells) has smooth muscle cell properties and participates in the milk ejection stimulated by oxytocin release [34,35]. The acini are organized in clusters, and each cluster forms a lobule (Figure 2A and Figure 3A) [28,32,36]. A lobule is formed by 10 to 100 acini (0.12 mm in diameter), a set of 20 to 40 lobules form a lobe, and 15 to 20 lobes form the glandular tissue [28,32]. Each acinus in a lobule drains into an intralobular terminal duct and this duct drain is, in turn, connected by extralobular terminal ducts [32]. The structure composed of a lobule associated with intralobular and extralobular terminal ducts is called a terminal duct lobular unit (TDLU), and constitutes the morphofunctional unit of the mammary gland [16,37]. The terminal ducts drain into the subsegmental and segmental ducts, with the latter also being known as lactiferous ducts [37]. Each lactiferous duct receives several mammary ducts, and has a dilation close to the nipple, named the lactiferous sinus. The end of each lactiferous sinus terminates in a nipple opening. There are 10–25 small opening sites arranged in a ring on the surface of the nipple through which milk is expelled. The nipple is a raised area of modified skin, surrounded by an areola, which contains large sebaceous units that form small nodular elevations (Montgomery’s tubercles) [38]. Furthermore, the nipple is composed of smooth horizontal and longitudinal muscle fibers that are related to the nipple base [39]. Both nipple and areola show increased melanin pigmentation after the first pregnancy [38]. The glandular tissue and ducts are surrounded by adipose tissue and supported by suspensory ligaments (known as Cooper’s ligaments)—a loose structure of dense fibrous connective tissue [28].

As already mentioned, the development of the mammary glands in women begins at puberty. At that time, TDLU structures have not yet been differentiated, and are called terminal end buds (TEBs). TEBs are composed of two morphologically distinct cells. The “body cells”, which are centrally organized into multiple layers, and the cap cells, which form a simple layer that surrounds the body cells. The cap cells and body cells differentiate into myoepithelial and luminal cells, respectively [40,41].

**Figure 4:**
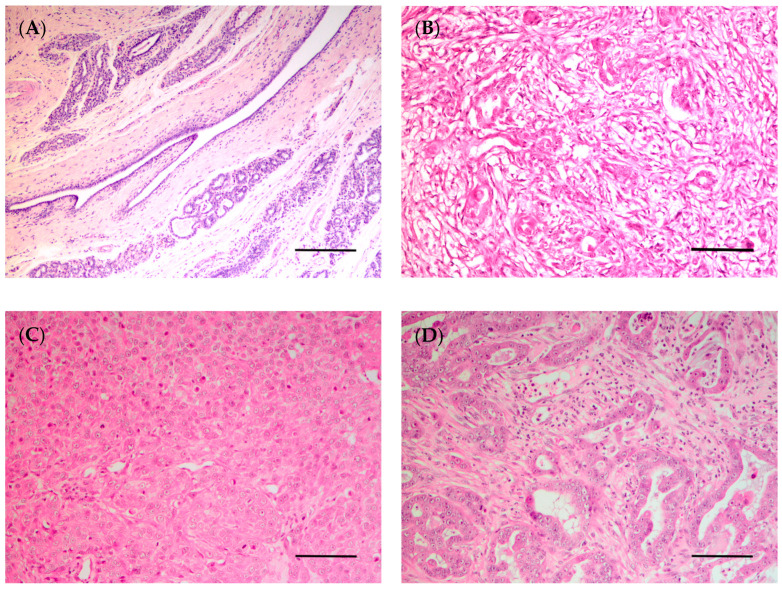
Canine mammary gland. (**A**) Normal mammary gland (bar = 200 µm); (**B**) complex carcinoma (bar = 100 µm); (**C**) solid carcinoma (bar = 100 µm); (**D**) invasive tubulopapillary carcinoma, high grade (bar = 100 µm). Hematoxylin and eosin staining.

The mammae of female dogs, similar to in woman, have a tubuloalveolar structure embedded in fibrovascular and adipose tissue (Figure 2B and Figure 4A) [42]. The branching system begins in the secretory alveoli and drains into the intralobular ducts, then into extralobular ducts, and finally into large lactiferous ducts. The large lactiferous ducts end in a lactiferous sinus, which continues into the nipple sinus and opens onto the nipple surface via the papillary ducts [42,43,44]. As in women, but fewer in number, each nipple has between 6 and 16 (up to 22) papillary duct orifices. The number of openings (papillary duct orifices) is determined by the number of sprouts present in the mammary gland. Each one of these ducts forms a lobe of the mammary gland and acts as an independent functional unit within the gland. The central papillary ducts tend to form an irregular design, while the peripheral ducts exhibit a ring shape [42,43,44,45,46]. A circular smooth muscle sphincter surrounds the teat ducts, which are lined by stratified squamous epithelium [46]. The teats in each mammary complex are conical, and somewhat thinner in the lateral direction, but the shape varies among breeds. The hair on the skin around the mammary gland is less dense, and the outer section of the teat is covered by epidermis that is slightly thicker than the epidermis of the adjacent skin [47].

Larger extralobular terminal ducts (lactiferous ducts) consist of a bilayer of luminal epithelium cells subtended by myoepithelial cells. Smaller distal extralobular and intralobular ducts are lined by a single layer of luminal epithelial cells with an outer layer of discontinuous myoepithelial cells [46,47]. Similar to women, secretory alveoli—which develop after hormonal stimulation—are composed of an inner layer of luminal epithelial cells, with some intracytoplasmatic lipid droplets, surrounded by an outer layer of myoepithelial cells, which in turn are surrounded by a basement membrane [42,46,47]. As in women, prolactin stimulates the gland to produce milk, and oxytocin allows milk to be ejected into the duct [48]. At birth, only the large ducts are formed. As in women, mammary development in dogs only begins at puberty, when the ovaries start releasing estrogens. Cell proliferation occurs at the terminal ends of the ducts to form TEBs. During pregnancy, the ducts develop and give rise to lobules and alveoli (lobuloalveolar unit) due to high levels of progesterone. The prolactin acts in presecretory alveolar cells that then differentiate into secretory alveolar cells. At parturition, the mammary gland can be described as a secretory ductal–lobular–alveolar structure. Alveolar regression starts 10 days postpartum, and is completed after approximately 40 days [44,46].

**Figure 5:**
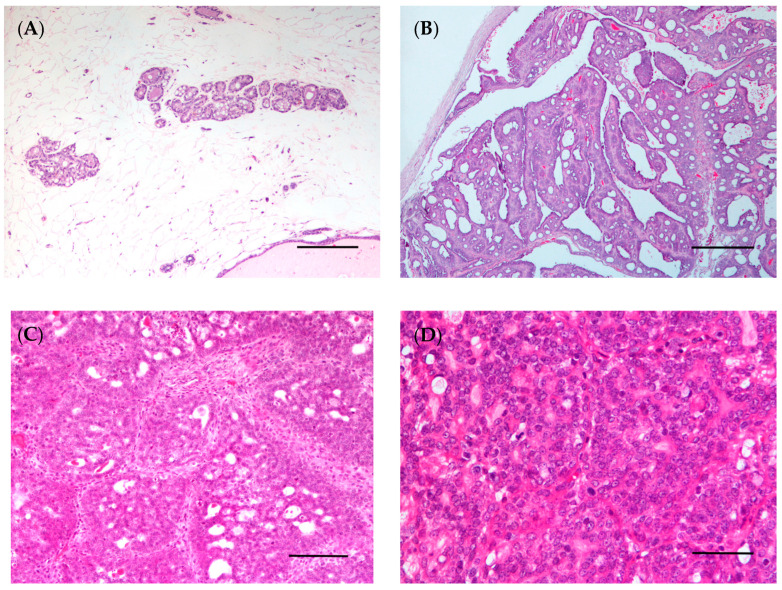
Rat mammary gland. (**A**) Normal mammary gland (bar = 100 μm); (**B**) intraductal papillary carcinoma (bar = 500 μm); (**C**) invasive cribriform carcinoma (bar = 100 μm); (**D**) invasive carcinoma, high grade (bar = 50 μm). Hematoxylin and eosin staining.

Histologically, the mammary glands of female rats have a tubuloalveolar morphology composed of a highly branched system of ducts and terminal secretory alveoli arranged in lobules, similar to that described for both women and female dogs (Figure 2C and Figure 5A). Each mammary gland has a single lactiferous duct that drains milk into the nipple, in contrast to what was described for humans and dogs, who have multiples lactiferous ducts (10–25 and 7–16, respectively) [20]. The lactiferous ducts of rats are composed of 5–10 secondary collecting ducts [16,20]. The ducts and lobules are similar to those of women and female dogs, and are embedded in adipose tissue, called mammary fat pad [49]. Luminal and myoepithelial cells have the same function and are stimulated by the same hormones, prolactin and oxytocin, respectively [49]. The number of TEBs reaches its maximum at 20 days of age. During pregnancy, an enlargement of the epithelium is observed, with a growth of lobules and ducts. Prolactin is the hormone responsible for alveologenesis [5,50].

### 3.1. Supply and Venous Drainage

In women, the breast receives blood supply through the internal, superior and lateral thoracic arteries, the acromiothoracic artery, the thoracodorsal artery and the lateral branches of the posterior intercostal artery. Most of the blood is supplied by the internal and lateral thoracic arteries [51]. The upper and outer portions of the breast are supplied by the lateral thoracic artery [52]. The venous drainage system is parallel to the arterial supply and adopts similar names [51]. The veins are divided into two subgroups—deep and superficial. The deep veins drain into internal thoracic, lateral thoracic, axillary, and upper intercostal veins, and the superficial veins drain into internal thoracic vein [52].

Mammary glands of female dogs are highly vascularized. The M1 and M2 glands receive arterial blood via the internal thoracic artery, through secondary branches of intercostal and lateral thoracic arteries. The M3 gland is supplied by the cranial superficial epigastric artery. Caudal abdominal and inguinal glands (M4 and M5) receive blood from caudal superficial epigastric and external pudendal arteries. The dog’s veins are mostly parallel to the course of the arteries, with the venous drainage being similar to the arterial supply, although small veins may cross the midline between the left and right mammary glands. The cranial and caudal superficial epigastric veins are the main veins of the mammary glands. The thoracic mammary gland drains into the cranial superficial epigastric vein, while the abdominal and inguinal mammary glands drain into the caudal superficial epigastric vein [15,46].

Several arteries supply the mammary glands of female rats. The thoracic region is supplied by the superficial cervical, internal and external thoracic, and axillary arteries, while the abdominal–inguinal region is supplied by the iliolumbar, superficial epigastric and external pudendal arteries [18].

There is no relationship between normal anatomical vascularization of the mammary gland in the female dogs with cancer development. According to the literature and our experience in this field, tumors are more frequent in the more caudal and denser mammary glands.

### 3.2. Lymphatic Drainage

The lymphatic system of the mammary gland is a diffuse, variable and extensive network of nodes and lymphatic vessels that receive lymph from the mammary gland [53,54]. The lymphatic system ranges from large vessels to lymphatic capillaries, and is abundant in the connective tissue of the alveolar lobule [50]. This system can be divided into two subgroups: superficial lymphatic (the drain skin over the mammary gland, except for the areola and nipple) and deep lymphatic (the mammary gland, as well as, the areola and nipple) [51,54]. In woman, the main sites of lymphatic drainage are axillary and internal mammary lymph nodes, with the axillary lymph nodes draining approximately 75% of mammary gland lymph [51,54]. The interpectoral, internal thoracic, supraclavicular, and infraclavicular lymph nodes are additional drainage areas [52]. The axillary nodes are usually divided into three levels (I, II and III) depending on their relationship to the pectoralis minor muscle. The level I nodes are located in the low axilla, lateral to the axillary border of pectoralis minor muscle. The level II nodes lie between the medial and the lateral borders of the pectoralis minor muscle—in the mid axilla —and they comprise the central nodes and subclavian nodes. Level III nodes are located in the apex of the axilla between the upper border of the pectoralis minor muscle and the lower border of the clavicle and include subclavicular nodes [52,55].

The lymphatic system is also essential for providing insight into predictive associations with metastatic risk. As in women, the lymphatic system in female dogs is considered a major route of metastasis from mammary tumors. Tumor cells may enter the lymphatic vessels and spread to regional lymph nodes. The first lymph node that receives lymph flow from the primary neoplasm is referred to as the sentinel lymph node and is often used to guide surgical treatment, as an indicator of disease progression and a prognostic marker [56,57]. Additionally, anti-lymphangiogenic strategies can be developed for the prevention and treatment of metastatic disease [58,59].

In female dogs, both cranial (M1) and caudal thoracic (M2) mammary glands drain primarily into the axillary lymph center, but they may also drain secondarily into the superficial cervical nodes. The drainage of the cranial abdominal gland (M3) is inconsistent, draining into the axillary lymph nodes (most of the lymph), but also into the superficial inguinal lymph nodes, if it enters the caudal abdominal gland lymphatics [15,60]. The caudal abdominal (M4) and inguinal (M5) mammary glands drain into the superficial inguinal lymph nodes and medial iliac nodes (secondary). Despite being a rare process, the M2 can drain simultaneously into the superficial inguinal and medial iliac nodes [60]. In 2003, Pereira and colleagues reported that M4 and M5 glands could also drain lymph into the superficial popliteal lymph nodes. The M1, M2 and M3 glands have lymphatic communications with each other, whereas the M4 gland only communicates with the M5 gland [61]. There are no direct connections between the left and right mammary lymphatic vessels [15].

The lymphatic drainage of the female rat‘s mammary glands, like in female dogs, is dependent on the position of the mammary glands. Lymphatic drainage from cervical mammary glands occurs into the proper axillary, accessory axillary and the superficial cervical lymph nodes. The cranial thoracic mammary gland drains into the proper axillary and the accessory axillary lymph nodes, whereas the caudal thoracic mammary gland only drains into the proper axillary lymph node. Abdominal and inguinal mammary glands drain into the proper axillary and inguinal lymph nodes [62].

In this context, it is crucial to understand the lymphatic system for metastatic study and to collect samples to perform other studies namely in vitro studies.

### 3.3. Innervation

The innervation of the breast consists of nerves containing sympathetic and sensory efferent fibers. Most sensory fibers end close to the epidermis of the nipple, suggesting that they play a role in the afferent pathway of the milk ejection process that is signaled to the central nervous system [63]. The sensory nerves mediate nociceptive and tactile sensations, whereas vascular sympathetic nerves subserve thermoregulatory roles. Sympathetic nerves, which mainly innervate arterioles, release norepinephrine and neuropeptide Y, and may promote lactation by contracting smooth muscle and myoepithelial cells. This contraction can promote milk secretion and mitigate its production by vasoconstriction, which consequently reduces the blood flow to the alveoli needed for milk production. The sensory nerves can release neuropeptides such as substance P, calcitonin gene-related peptide, and adenylate cyclase-activating polypeptide that prevent milk flow. This process can be inhibited either by relaxing the ductal contractile cells or by increasing the blood flow, thus promoting plasma filtration and increasing milk production [64]. The mammary gland in women is innervated by the lateral cutaneous branches of the third to sixth intercostal nerves, the anterior cutaneous branches of second to sixth thoracic intercostal nerves and the supraclavicular nerves [65]. Nipple nerve supply derives primarily from the anterior and lateral cutaneous branches of the fourth intercostal nerve, with additional innervation by cutaneous branches of the third and fifth intercostal nerves [52,66].

In female dogs, the mammary gland is innervated by branches of the intercostal and genitofemoral nerves. The cranial thoracic mammary gland receives its nerve supply from the lateral cutaneous branches of the fourth, fifth, and sixth thoracic ventral nerves (intercostal). The caudal thoracic gland is enervated by lateral cutaneous branches of the sixth and seventh thoracic ventral nerves (intercostal). The abdominal and inguinal mammary glands are innervated by the genitofemoral nerve and the ventral cutaneous branches of the first three lumbar nerves (cranial iliohypogastric, caudal iliohypogastric, and ilioinguinal) [15].

In female rats, sympathetic and sensory innervation is present within the nipple and mammary gland, with sympathetic nerves being the most prominent nerve type [64]. No studies were found in the literature specifying the innervation of the mammary gland in rats. The main features of mammary glands in women, female dogs and female rats are outlined in Table 1.

Recent evidence has shown that tumors can recruit peripheral nerves to the tumor microenvironment that are implicated in proliferation, invasion, metastasis, and immune evasion, resulting in increased tumor growth [67,68,69]. For example, nerve fibers can release signaling molecules such as nerve growth factors, which can increase the survival and growth of tumor cells. This process, called “perineuronal invasion”, is associated with an aggressive phenotype and poor prognosis [67,68,69,70]. A recent study demonstrated that a poor prognosis is associated with increased sympathetic and decreased parasympathetic nerve density in tumors, suggesting that sympathetic nerves accelerated breast cancer progression [71]. Like the lymphatic systems, knowledge of the nervous system is vital to understand tumor behavior and should be taken into consideration when are collected samples to perform in vitro studies.

## 4. Breast/Mammary Cancer

### 4.1. Epidemiology

Cancer rates have increased in humans and domestic animals, making this disease one of the leading causes of death worldwide in these species [72]. The access to vaccines, better nutrition and veterinary care, as well as a greater interest of pet owners are allowing dogs to live longer, which contribute to the increase in cancer cases in company animals [73]. Mammary tissue is particularly sensitive to carcinogenesis because it undergoes several changes during the female’s life span (puberty, pregnancy, lactation and, in women, menopause), which are mediated by different growth factors and hormones [74]. Mammary neoplasms are one of the most prevalent types of cancer in humans, dogs and cats, but are rare in other species [75]. Due to the interspecies biological and anatomical differences, the terminology used to describe this disease varies from “breast cancer” or “breast tumor” in humans, to “mammary cancer” or “mammary tumor” in non-humans [21].

In women, breast cancer is not only the most frequently diagnosed type of cancer, it is also the type of cancer that is responsible for the most deaths [76,77]. According to the World Health Organization (WHO), more than 600,000 women died from breast cancer worldwide in the year 2020, and there were approximately 2.2 million new breast cancer cases. In the same year, breast cancer accounted for about 15.5% of all cancers and 24.5% of all cancers in women [77]. Indeed, one in every 10 new cancer diagnoses per year is breast cancer. This cancer has a high incidence in North America, Europe and Oceania [77]. Breast cancer is more common in the left breast, and breast cancer metastases are mainly found in the bone, followed by the liver, and then the lung and brain [78,79]. However, the most common place to which breast cancer spreads is to the axillary lymph nodes [80].

Mammary neoplasia is one of the most common tumors in intact adult female dogs and in advanced-aged sterilized female dogs [10,81,82]. Canine mammary tumors (CMTs) represent almost 50% of all canine neoplasms [83]. According to large European databases of cancer registries, the incidence of mammary gland tumors ranges from 111 per 10,000 to 200 per 100,000 dog-years at risk, with the average age of first diagnosis being roughly 7 years of age [84,85]. Salas et al. (2015) reported that the annual incidence of mammary tumors was 16.8%, with benign and malignant tumors presenting similar frequencies (47.7% and 47.5%, respectively) [9]. These data corroborate another study conducted by Canadas et al. (2019) [86]. The prevalence varies by geographic location, being lower in countries where ovariectomy is routinely performed [87]. According to Santos et al. (2020), tumors are frequently found in the caudal abdominal (M4) and inguinal (M5) mammary glands (27.45% and 32.67%, respectively) [88], which is in agreement with another study performed by Nguyen et al. (2018) [89]. Fifty to seventy percent of dogs have multiple mammary tumors [90]. Malignant CMTs can metastasize and eventually become fatal [9]. Similar to in women, the presence of metastases can initially be observed in the lymph nodes, notably in the inguinal or axillary lymph nodes spread through the lymphatic system. In addition, metastasis can spread hematogenously and reach the lungs or more distant body sites, including the liver, spleen, heart and bone [56,81,91].

In female rats, spontaneous mammary tumors are the second most frequent neoplasm after the pituitary gland tumors [5]. Like in men and male dogs, male rats are less affected by spontaneous development of mammary tumors than female rats. Spontaneous mammary tumors develop in 0.5–16% of intact males, and 30–90% of intact female rats (depending on the strain) [92]. There are several strains of rats that can develop spontaneous mammary tumors, including August, Albany-Hooded, Copenhagen, Fisher, Lewis, Osborne–Mendel, Sprague–Dawley, Wistar and Wistar/Furth [21]. The incidence of spontaneous mammary tumors in female Sprague–Dawley rats ranges from 30 to 67% [93]. However, these spontaneous tumors are mainly observed in older animals. Researchers have previously described that ovariectomy before 5–7 months of age decreases the incidence of mammary cancer by 95% when compared with non-ovariectomized rats [94]. The development of mammary tumors in rats before one year of age is uncommon, and the incidence increases after 18 months of age [95]. Considering the low incidence of spontaneous mammary tumors during the first year of life, female rats are frequently used as models of chemically induced mammary tumors.

With respect to chemically induced mammary cancer, female Sprague–Dawley and Wistar rat strains are more susceptible to chemical carcinogens than the Fischer strain [20]. In addition, nulliparous female rats are more susceptible to developing carcinomas due to incomplete differentiation of the gland at the time of carcinogen administration, since there is a reduction in the number of undifferentiated structures after pregnancy and lactation. For the same reason, the susceptibility of the female rat mammary gland to chemical carcinogens decreases with age [96,97]. To obtain a high mammary cancer induction rate, the carcinogen should be administered at between 45 and 60 days of age, which coincides with animals’ sexual maturity and high proliferation index of pluripotent cells from the terminal end buds of the mammary gland [74]. Indeed, 100% of female rats exposed to the carcinogen N-methyl-N-nitrosourea (MNU) developed mammary tumors 23 weeks after carcinogen exposure [98]. The MNU and 7,12-dimethylbenz[a]anthracene (DMBA) are the most frequently used carcinogens, and the spectrum of induced lesions varies from benign to malignant lesions [21,99]. There are studies indicating that MNU-induced mammary carcinomas are more aggressive and have a worse prognosis when compared with those induced by DMBA [100,101]. Unlike animals with spontaneous tumors, where the information is not clear, several studies with chemically induced female rat mammary tumors have described that tumor incidence is higher in thoracic region and in right mammary chain [101,102]. In summary, multiple factors, including gender, genetic background, age and parity, influence the susceptibility of rat mammary glands to the carcinogens MNU and DMBA [103]. Although also susceptible to these carcinogens, mice are not frequently used as models of chemically induced mammary cancer, probably due to their small size.

### 4.2. Risk Factors and Breast Cancer Development

Age, sex, race, reproductive factors, parity, family history, breast density, obesity and lifestyle are some of the factors that influence the risk of breast cancer development in women [104]. Breast cancer development rates increase with age, and older women consequently have a higher risk of developing cancer, with the mean age of diagnosis being 62 years [105]. Several studies have shown an association between advanced age at menopause (over 50 years) and an increased incidence of breast cancer [106,107,108]. Another factor associated with breast cancer risk is pregnancy, making it necessary to take into account the age of first calving. Women who had their first calving at the age of 30 or younger tend to receive a general protective benefit from pregnancy [109,110,111]. Nulliparous women are associated with a higher risk of breast cancer than parous women due to the higher number of ovulatory cycles [112]. Another reason for this is the fact that breastfeeding supports the differentiation of mammary cells, and differentiated cells are less likely to become cancerous. Contradictorily, there is a study that observed that multiple births increased the risk of developing breast cancer [110]. With respect to hormonal contraceptive use, there is a small increase in the risk of breast cancer among women who use it compared to those who have never used hormonal contraceptives [113,114]. Curiously, it was recently reported that this risk decreases after the discontinuation of hormonal contraceptives [115]. Similarly, numerous prospective studies have found an increased risk of breast cancer in postmenopausal women who use hormone replacement therapy for a long period of time [116,117,118]. Additionally, bilateral ovariectomy before 45 years of age can reduce breast cancer risk [119]. Obesity is associated with a higher risk of breast cancer, which increases after menopause, as adipose tissue acts as the main reservoir of estrogen [120]. Additionally, consumption of red meat and processed meat was significantly associated with an increased risk of breast cancer [121]. With respect to genetic susceptibility, approximately 30% of breast cancer risk is hereditary [122], and most familial cases of breast cancer in women have been associated with mutations in BReast CAncer (BRCA) genes 1 and 2, accounting for 5–10% of breast cancer cases [123]. The average risk of developing breast cancer with the BRCA1 mutation at age 70, is 65% [124]. Other genes such as *ATM, BARD1, CDH1, CHEK2, PALB2, PTEN, RAD51C, RAD51D, STK11 and TP53* have also been associated with a risk of developing breast cancer [125]. As expected, lifestyle might also influence the risk of breast cancer development. Alcohol consumption [126,127,128], smoking [129], physical inactivity [130] and hypercaloric diet [128,131,132,133] are factors that have been associated with an increased risk of breast cancer. The controversial results for some factors such as blood group, age of menarche, abortion, ovulation-stimulating drugs, breast density, coffee consumption and duration of sleep, do not allow the establishment of a direct association with the risk of breast cancer development [111].

Age-wise, CMTs occur frequently in middle-aged to older female dogs with an increased risk between 7 and 13 years of age [44,82], with the development of malignant neoplasms occurring more frequently in older female dogs [90]. Indeed, the average age at diagnosis is approximately 10.5 years, which is equivalent to a 65.5-year-old woman, suggesting that the age of disease onset is one of the epidemiological factors that female dogs and women share [134]. Malignant mammary tumors are usually diagnosed between 9 and 11 years of age, while benign neoplasms are diagnosed at an average age of 7–9 years. Malignant neoplasms are rare in dogs under 5 years of age [87,90]. It is important to closely consider the peak occurrence of cancer based on age, taking into account the fact that larger dog breeds have a naturally shorter lifespan, and therefore are diagnosed with cancer at a younger age than smaller breeds [87]. Similar to women, nulliparous dogs have a higher risk for developing CMTs than multiparous [135]. The risk of developing CMTs also differs according to the age/period of the ovariohysterectomy (spaying/neutering) [136]. The risk is 0.05% if the female dog was spayed before the first estrous cycle, increasing to 8% and 26% if the female dog was spayed after the first or second estrous, respectively [137]. Exposure to hormones (progestins and estrogen) can also increase the risk of developing mammary tumors. In addition, the use of low-doses of progestin tend to induce benign tumors while the combined use of progestin and estrogen increases the risk of malignant tumors [87]. Similar to in women, mutations in the BRCA1 and BRCA2 genes have been linked to a four-fold increased risk of mammary tumor development in dogs [138]. Additionally, in female dogs, too, obesity or overweight, mainly between 9 and 12 months of age, has also been associated with increased risk of tumor development [82,139]. One study showed that having a thin physique reduced the risk of mammary cancer among non-spayed dogs by 40% and spayed dogs by 99% [140]. In addition, dogs on a homemade diet with high-red meat portions was associated with a higher risk of developing mammary tumors and dysplasia compared to a commercial diet [141]. CMTs tend to be more common in pure breeds when compared to animals of mixed breed [87,142]. However, Pastor and co-workers reported that mixed breeds are more likely to suffer from mammary tumors than pure breeds [10]. As far as size is concerned, smaller pure breeds are more often affected by mammary tumors [46,87]. Contrary to this information, previous evidence indicates that small breeds are the least predisposed for mammary cancer development [143]. In the small breeds category, Chihuahuas, Dachshunds, Yorkshire Terriers, Maltese, and Cocker Spaniels are high-risk dog breeds. With regard to larger breeds, those at high risk include Boxers, Brittany Spaniels, Dobermans, English Springer Spaniels, English Setters, German Shepherds, and Pointers. These differences in the risk of developing mammary cancer suggests a genetic influence on canine mammary tumorigenesis [9,42,44,46,81,87]. Spontaneous mammary tumors in female rats are very rare, and are mostly described in older animals, as mentioned above. Although the rat is an exotic pet, there have been no studies addressing the causes and risk factors for mammary cancer development in these animals, but we can assume that age is one of them.

### 4.3. Histological and Molecular Classification of Breast/Mammary Tumors

Histopathology allows histological diagnosis, enabling the determination of several parameters, such as tumor histotype, histological grade, and lymph node involvement [144]. Histologically, breast cancer can be categorized into preinvasive carcinoma (or in situ) and invasive carcinoma (Figure 3B–D) [144,145]. In situ carcinoma is confined to the basement membrane, and can be sub-categorized as either ductal in situ carcinoma, which is the most prevalent type of in situ carcinoma (80–90%), or lobular in situ carcinoma [146,147]. Currently, the WHO classifies breast carcinoma into 19 different major histological subtypes. The most frequently diagnosed subtypes include invasive ductal carcinoma (recently renamed invasive carcinoma of no special type, IC-NST) and lobular carcinoma, accounting for 70–75% and 10–14% of all cases, respectively. The carcinomas of special type include 17 rare subtypes: tubular carcinoma, cribriform carcinoma, and mucinous carcinoma, all of which are associated with good prognosis; and pleiomorphic lobular carcinoma, high-grade metaplastic carcinoma, micropapillary carcinoma, and inflammatory breast cancer, which are associated with poor prognosis [144].

In addition to histological classification, breast invasive carcinomas can be grouped according to their histological grade using the Elston–Ellis modification of the Scarff–Bloom–Richardson grading system, also known as the Nottingham Grading System [148]. This grading system is a well-established prognostic factor for breast cancer, and is widely used in clinical decision making. It is composed of three pathological findings: degree of tubular formation, nuclear pleomorphism, and mitotic count; and each category receives a score from 1 to 3, for a total score ranging from 3 to 9 [149]. Tubule formation is scored 1 when the tubules comprise >75% of the tumor, scored 2 when they comprise 10–75% of the tumor, and scored 3 when they comprise <10% of the tumor. The nuclear pleomorphism is scored 1 for small uniform regular cells, 2 for moderate variation in nuclear size and shape, and 3 for marked variation in nuclear size. With respect to the mitotic index, mitoses are counted in 10 high-power fields (40×) and scored 1 if between 0 and 10 mitoses are observed, scored 2 if 11–19 mitoses are observed, and scored 3 if >20 mitoses are observed. The sum of the three scores is used to grade each tumor as follows: grade I (well-differentiated, or low-grade; 3–5 points); grade II, (intermediate, moderately differentiated; 6–7 points); and grade III (high grade, poorly differentiated; 8–9 points) [150].

Breast tumors are also categorized into five stages (from 0 to IV) according to the tumor, node, metastasis (TNM) staging system. This system was developed by Pierre Denoix in the 1940s and 1950s, and takes tumor size, lymph node involvement and metastatic progression into consideration [151,152]. This system is used to determine the stage of the disease, also helping in prognosis and therapeutic approach [152]. In 2018, the most recent edition of the AJCC Cancer Staging Manual, the eighth edition, was globally adopted. This edition includes two staging systems: (1) the anatomic stage, which includes the size of the primary tumor, nodal status, and distant metastasis; (2) the prognostic stage, which includes tumor grade, hormone receptor and oncogene expression [152]. High histological grade and high stage of the disease are associated with worse prognosis and lower survival time [148,153,154].

In breast cancer, immunohistochemistry is routinely performed to assist with the prognosis and determine the specific treatment for each patient [155]. The breast carcinomas immunophenotype is determined by using biomarkers such as estrogen receptor (ER), progesterone receptor (PR), human epidermal growth factor 2 (HER2) and Ki-67 proliferation marker, which constitute prognostic factors and are important predictive factors for hormonal and anti-HER2-targeted therapy [156]. Lastly, based on molecular aspects, breast cancer can be categorized into five clinical subtypes: luminal A (ER^+^, PR^+^, HER2^−^, low Ki-67 index), luminal B HER2^−^ (ER^+^, PR^+^, HER2^−^, high Ki-67 index), luminal B HER2^+^ (ER^+^, PR^+^, HER2^+^, high Ki-67 index), HER2-enriched (ER^−^, PR^−^, HER2^+^), and triple-negative breast cancer (TNBC; ER^−^, PR^−^, HER2^−^, high Ki-67 index) [151]. The TNBC positive for basal markers, such as cytokeratins 5 or 6, is referred to as the basal-like subtype of breast cancer. Most of TNBCs (75–85%) are of the basal-like subtype, and therefore there is an overlap in the terms, which causes a significant loss of distinction [157,158].

A study published in 2020 analyzing 740 breast cancers in women found that 58.5% of the tumors were luminal A, 16% were triple-negative, 14% were luminal B, and 11.5% were HER2-enriched. This study also concluded that the majority of luminal A breast cancers were lobular carcinomas, and the triple-negative and HER2-overexpression cancers had higher histological grade and larger size [159]. Indeed, luminal A subtype is the most frequent subtype, corresponding to about 60–70% of invasive breast tumors, and is associated with low histological grade and favorable prognosis [160,161]. Luminal B subtype accounts for about 15–20% of invasive breast tumors, has a higher proliferation index (Ki-67 greater than 14%) and is associated with higher histological grade and worse prognosis, when compared to luminal A [144,160,161]. Moreover, women with luminal B tumors are often diagnosed at a younger age than those with luminal A tumors [162,163]. Most luminal A and B cancers are estrogen dependent, and hormone therapy constitutes an effective approach for the treatment of these types of cancer [164]. HER2-enriched cancers represent about 15–30% of all breast cancers. Once they are strongly positive for HER2 and negative for hormone receptors, this subtype has poor prognosis when compared with luminal A and B subtypes, and anti-HER2 therapy is an adequate therapeutic approach for this subtype [165,166,167,168]. The TNBC subtype is so named due to the non-expression of ER, PR and HER2, representing about 10–20% of breast cancer cases, and is the subtype with the worst prognosis [169,170]. TNBC occurs more frequently in women under the age of 40 and with mutations in the *BRCA1* gene [171], and is not responsive to hormone therapy or anti-HER2 approaches [169].

In 1974, the WHO published the first “International Histological Classification of Tumors of Domestic Animals”, which was later modified in 1999 [172]. In 2011, a new histological classification was proposed by Goldschmidt et al. [172], which was applied in routine veterinary diagnostic pathology. Although most of the studies conducted in CMTs are based on the latter classification, in 2019, a new histopathological classification for CMTs was published by the Davis–Thompson Foundation [47]. Since then, some studies have used this novel classification to categorize the neoplastic lesions in canine mammary glands [173,174,175,176,177,178,179]. This new classification classifies epithelial neoplastic lesions as either benign or malignant, which are further subdivided into simple, non-simple and ductal-associated tumors. Most tumors in dogs are non-simple, being associated with the proliferation of both luminal and myoepithelial cells (Figure 4B–D) [47]. Special types of malignant epithelial tumors include seven entities: squamous cell, adenosquamous, mucinous, lipid-rich and spindle cell carcinoma, malignant myoepithelioma and inflammatory mammary carcinoma [47].

The histological grading system used worldwide for malignant CMTs was proposed by Peña et al. (2013), and is an adaptation of the Nottingham method utilized for human breast cancer [148,180]. Therefore, lesions are classified according to the same criteria (tubule formation, nuclear pleomorphism and mitotic index) and also categorized into three grades (grade I, II or III) [180]. Similar to humans, CMTs can be categorized into five stages (from I to V), according to the modified TNM staging system, where stages IV and V are considered to be advanced clinical stages [181]. This is a simpler model when compared to the human breast cancer model, where stage I corresponds to a primary tumor size of less than 3 cm, stage II to tumors between 3 and 5 cm, and stage III to tumors more than 5 cm. Regardless of tumor size, the presence of lymph node metastasis is considered stage IV, and the presence of distant metastasis is considered stage V. This staging system should only be applied to epithelial tumors (non-inflammatory) and not to sarcomas [87].

A study conducted by Burrai and colleagues in CMTs showed that 46.5% were benign tumors and 53.5% were malignant. Of the 1866 single mammary neoplasms analyzed, 12.8% were classified as simple benign tumors, 33.6% as non-simple benign tumors, 33.4% as simple malignant, 18.27% as non-simple malignant, 1.34% as special-type malignant and 0.5% as sarcomas. Among the benign lesions, the most frequent were mixed tumors (37.95%), complex adenomas (32.41%) and simple adenomas (25.9%), while the most frequent malignant tumors were simple tubulopapillary (25.60%) and complex carcinomas (24.92%). Regarding the grade, the majority of the malignant tumors were of grade I (83.7%), followed by grade II (14.81%) and grade III (1.4%) [173].

As in women, CMTs can also have a molecular classification. However, in the clinical setting, immunohistochemistry is not frequently performed due to the high cost.

Some studies in CMTs use molecular classifications of human breast cancer to classify CMTs, and hypothesize that CMTs are also a good model for studying immunophenotypes [3,134,182,183]. Another problem associated with classification of CMTs is the use of different immunohistochemical biomarkers and classification criteria, leading to contradictory and inconclusive results [184,185]. Depending on the study and the antibodies used, the following molecular subtypes of CMTs can be found: luminal A, luminal B, HER2 overexpressing, triple-negative basal-like and triple-negative normal-like [186]. The difference between triple-negative and basal-like subtypes was evidenced in several veterinary studies, and is associated with very distinct survival times in dogs [3,182,187,188]. The triple-negative cancer has been associated with shorter survival times [3]. More malignant tumors in both humans and dogs are more often ER and PR negative.

Abadie and colleagues (2018) evaluated the immunophenotype of 350 CMTs and categorized 50 tumors as luminal A (14.3%), 33 as luminal B (9.4%) and 267 as triple-negative (76.3%), with 205 (58.6%) triple-negative basal-like and 62 (17.7%) triple-negative normal-like [3]. No HER2 overexpression was observed. In contrast, in addition to 42 luminal A tumors (38.2%), 41 luminal B tumors (37.3%) and 17 triple-negative tumors (15.4%), 10 HER2-positive tumors (9.1%) were also observed in the other group [189]. Other studies have also observed HER2-overexpressing subtype in CMTs, supporting the finding of Varallo et al. (2019) [182,186].

Although most of the studies show that female dogs have a higher prevalence of mammary tumors of the basal-like and triple-negative subtypes [3,190], there is no consensus regarding these results [189,191]. These subtypes are associated with an aggressive phenotype, a significant association with metastasis, worse TNM stage and shorter overall survival and disease-free interval when compared with other immunophenotypes [3,182,186,192,193]. Thus, these findings suggest that CMTs could be used as spontaneous models to investigate human triple-negative and basal-like breast carcinomas [3,182]. The molecular subtypes are strongly correlated with histological grade, histological type and survival [182,194]. In sum, the luminal A phenotype is associated with grade I and II carcinomas, while the basal-like subtype is linked with grade III; complex carcinomas more frequent belong to the luminal A phenotype, and the basal-like phenotype is associated with simple carcinomas, mostly solid carcinomas [46].

With respect to mammary tumors in female rats, the histological type may vary depending on whether the tumors are spontaneous or chemically induced. Fibroadenomas, followed by adenocarcinomas, are the most common spontaneous neoplasm of the rat mammary gland [20]. In a study using Wistar rats, fibroadenomas (36.1%), followed by adenocarcinomas (6.7%) and adenomas (3.9%), were among the most common spontaneous mammary neoplasms [195]. Similar results were found in another study using Sprague–Dawley rats, with the identification of fibroadenomas (19.0%), adenocarcinomas (8.8%), and adenomas (3.5%) [193]. The histological classification for chemically induced female rat mammary tumors was established in 2000 [196]. The female rat mammary tumors may be histologically classified as: epithelial neoplasms (subdivided in benign, precancerous and malignant lesions), stromal, epithelial–stromal and nonneoplastic lesions. Malignant lesions may be subdivided into non-invasive (or in situ) and invasive carcinoma; these can then be classified into five subtypes: papillary, cribriform, comedo, tubular carcinoma and adenoid cystic (Figure 5B–D) [97,196]. Using the rat model of mammary cancer MNU-induced, researchers classified the mammary lesions according to their histogenesis noting that the epithelial malignant lesions (89.19%) were the most common, followed by epithelial benign and preneoplastic lesions (both 5.41%) [197]. Another study observed that invasive papillary carcinomas were the most typical and frequent mammary tumor DMBA and MNU-induced in the rat, followed by invasive cribriform carcinomas, while the invasive comedo carcinoma represents the least observed lesion [97]. These data were corroborated by other studies [100,197]. Immunohistochemical assays have been performed in chemically induced mammary tumors in female rats to characterize the estrogen and progesterone receptors in these tumors, making them an adequate model for developing and testing new hormone therapies [98]. Despite this, the subtypes most frequently detected in chemically induced rat mammary tumors are luminal A and B [97]. For this reason, transplant models (such as cell-derived xenografts, patient-derived xenografts and syngeneic models) are the best choice for testing new therapies for molecular subtypes of breast cancer, such as HER2 overexpressing and triple-negative.

## 5. Advantages and Disadvantages of Spontaneous Tumors and Induced Animal Models for Studying Breast Cancer in Women

A model that is perfect for all studies does not exist, and even if a model is adequate for a particular study, it may not be suitable for exploring other hypotheses. Thus, depending on the objectives of each experiment, researchers should select the model that best suits the scientific question to be investigated. Clinically, CMTs share many similarities with breast cancer in women in terms of histopathology, biological behavior, hormone dependence, risk factors and genetic alterations [3,72,198,199,200]. Dogs often share the same environment as their owners, and in some cases exhibit similar co-morbidities [72]. For these reasons, female dogs are considered by many researchers to be an attractive model for studying spontaneous breast cancer. On the other hand, female dog tumors are also of interest for generating 3D in vitro models. In addition, female dogs develop tumors in a shorter time, due to their shorter longevity compared to humans, which may constitute an advantage [72]. Thus, the time required to collect the minimum number of dogs for a single study is shorter than in humans [134]. In contrast with women, female dogs may frequently be diagnosed with multiple mammary tumors, and this can cause difficulties in interpreting the results [201]. Another limitation is that many domestic dogs frequently undergo ovariohysterectomy at a young age, and this important point must be taken into account in comparative models [136,202]. Studies that use female dogs are reliant on pet owners for postoperative treatment in cases that do not require hospitalization and for follow-up, which constitutes a limitation for obtaining valid and homogeneous results [134]. Finally, due to the conditions required in animal facilities, including long periods of time and high costs, as well as for ethical reasons, female dogs are not used as experimental models [4].

To date, female rats are still by far the most commonly used animal models for breast cancer research [4,5,203]. Indeed, they have several advantages, namely their low cost, high reproducibility and specificity, small size, and easy accommodation and manipulation when compared to other animal models [4,5]. Additionally, their physiological and genetic backgrounds have been well characterized, and they are similar to humans in several respects [5]. Furthermore, the use of laboratory animals enables better follow-up. For example, following exposure to the carcinogen, tumor formation can be closely monitored on a weekly basis through manual palpation. After tumor diagnosis, this close follow-up allows several types of data to be obtained, such as overall incidence, latency and multiplicity (i.e., number of tumors per rat) [103]. At the end of the experiment, mammary tumors are collected, weighed, and stored/processed for various analyses, including histopathology [103] and omics-based analysis [204,205], among others. Lastly, the use of female rats as a mammary cancer model must be approved by the appropriate authorities for the protection of animals used for scientific purposes, and all legal aspects should be respected [5]. One limitation of using female rats in research is that their mammary tumors rarely metastasize, which could in part be due to the shorter follow-up time of most experiments with rats compared with human patients [57,97]. However, a recent study reported that two MNU-induced rats demonstrated lung metastases 35 weeks after carcinogen exposure [206]. Another study with MNU-induced mammary tumors in female rats found one animal with metastasis in a lymph node and one more with metastases in the liver and spleen [101]. Another limitation of laboratory models of breast cancer is the size of the tumors. Often the tumors are small in size, and it is not possible to collect samples from the same tumor to perform different techniques simultaneously. This is overcome by tumor samples from female dogs, because they are in most cases larger, and a spontaneous dog tumor sample can be used simultaneously for additional studies.

## 6. Spontaneous Tumors and Animal Models as a Source of Samples for Alternative 3D In Vitro Models

**Figure 6:**
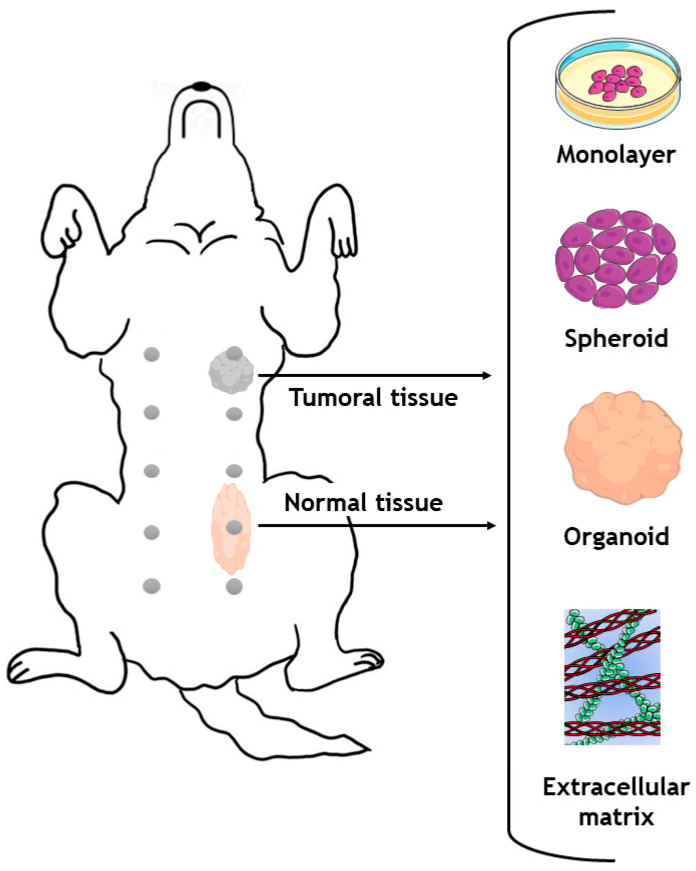
Generation of different in vitro models with cells or with extracellular matrix obtained from a single dog donor. Parts of the figure were drawn by using pictures from Servier Medical Art.

In addition to the histological, proteomic and molecular analyses that can be performed on tumor samples obtained from female dogs and female rats, these samples can also serve as a source for generating alternative in vitro models. The number of samples obtained from animals is sometimes not enough to perform the necessary assays to test the scientific hypothesis; therefore, alternative 3D in vitro tumor models could enable researchers to study different therapies in less time and at a larger scale, and could also make a contribution to the development of personalized therapies [207,208,209]. In addition, animal testing is often expensive, time consuming, and ethically controversial [210]. As a consequence, researchers are increasingly turning to alternative 3D in vitro models that can provide a more accurate reflection of human disease biology as means toreduce the reliance on animal testing [211,212]. In fact, 3D in vitro tumor models are increasingly considered to be an excellent alternative to animal studies [213,214]. In this context, one can also consider that spontaneous and/or induced animal models can also act as sources for cells to bioengineer 3D in vitro models from a single tumor sample, which opens up many possibilities in translational research (Figure 6) [215]. By using advanced technologies to manipulate cells, and at different length scales (i.e., 3D/4D bioprinting, mold casting, microfluidic technologies etc.), multiple replicates of the same tumor can be obtained for various assays, including drug screening [215,216,217]. In addition, animals can develop multiple tumors with different histopathological features and molecular subtypes. Thus, from a single animal, it may be possible to develop multiple models with different origins relevant for human breast cancer research [215].

The most common in vitro models for studying canine mammary tumors involve using immortalized cell lines grown in monolayers attached to a surface (2D models). Although these cell lines offer an abundant source of research material, they do not accurately represent conditions in a living organism, because they do not reproduce the natural spatial interactions between cells [218]. In recent years, the increasing complexity of *3D* in vitro models (e.g., spheroids/organoids, or organ-on-a-chip technologies) has contributed to the development of testing platforms that better recapitulate the tumor microenvironment found in vivo in humans. In this respect, it is recognized that 3D cell cultures are superior to 2D cultures in mimicking natural tissue physiology, cell–cell contacts, cell–matrix interactions and the different components of the tumor microenvironment, as well as major tumor hallmarks [219,220,221].

Spheroids are a type of 3D culture model and consist of a randomly assembled multicellular aggregate that can be generated from a variety of cell types, including cancer cells, and can also include stromal elements present in the breast tumor microenvironment [222]. They can be produced using various methods, such as hanging drop culture, hydrogel, rotary cell culture, or magnetic levitation methodologies, and can be maintained in culture for prolonged periods of time (>14 days) [223]. In addition, spheroids enable researchers to model in vitro key features of human tumors including 3D cell–cell contacts, proliferation, the existence of hypoxic regions, the formation of pH/nutrient gradients and nutrient deprivation, metastasis events, and interactions with immune system cells [224,225]. Spheroids are frequently employed as an initial strategy in the drug discovery process, allowing for high-throughput/high-content imaging analysis of the top-performing formulations before moving on to more complex and expensive animal models [226]. In addition to this, organoids, 3D structures, which are generally derived from primary cancer cells, represent another highly relevant class of in vitro model, since they closely resemble the histopathology/molecular sub-type, architecture and genetic background of the breast cancer tissues from which they were derived [227,228]. Organoids are typically generated by using a combination of growth factors and extracellular matrix (ECM) components that support cancer cell proliferation and self-assembly into highly ordered 3D living models. Many methods can be used to produce tumor organoids, including hanging drop, and culture in round-bottom non-adherent culture plates or hydrogel domes [229]. These models are generally cultured in ECM-mimetic synthetic (e.g., polyethylene glycol, polyvinyl alcohol and alginate) or ECM-derived natural (e.g., collagen, Matrigel^®^, hyaluronic acid) hydrogels [230,231]. In these approaches, ECM-derived/ECM-mimetic components are used to create a more physiologically relevant environment for cancer cells to proliferate [232,233]. The ECM-derived materials can generally be obtained through the decellularization of healthy or malignant tissues, with the latter being more valuable in the context of breast cancer modelling [234]. Studying malignant and healthy tissue allows researchers to study the differences between these tissues and to understand more precisely the mechanisms underlying tumorigenesis [235].

Although 3D in vitro models (i.e., spheroids or organoids) can be generated from artificially induced tumors in rats (including chemically induced, cell line-derived xenograft and patient-derived xenograft models), dogs have recently received particular attention, as they are spontaneous models. Recent studies have generated 3D in vitro models (spheroids and organoids) from canine mammary tumors that demonstrated similar features to the original tumor; these models make it possible to test several therapies [215,236]. In 2016, researchers developed 3D tumor spheroids in six-well Algi Matrix^TM^ plates from eight canine complex carcinoma and six canine simple carcinoma cell lines derived from canine tumors. The 3D spheroids were grown for two weeks, and both cell lines showed epidermal growth factor receptor (EGFR) expression. The authors also observed an upregulation of metalloproteinases (MMPs) 1, 3, 9 and 13, relaxin receptors 1 and 2 (*RXR1* and *RXR2*) and a downregulation of *CDH1* (E-cadherin) compared to the original tumor [236]. The results obtained using the 3D tumor spheroids were consistent with those obtained in vivo for canine tumors. This study demonstrated the great potential of 3D in vitro models and their potential in carrying out future research in which the tumor materials are procured from animals and then re-engineered in vitro [236]. In another recent study, 24 organoids derived from carcinoma, adenoma, and healthy mammary tissues from 16 dogs were generated [215]. The established organoids maintained the morphological and immunohistological characteristics of the primary tissue, as well as the hormone receptor status and genetic features. Furthermore, it was demonstrated that organoids from normal, benign and malignant tissues could be genetically modified using the CRISPR/Cas9 genetic engineering toolboxes, and the library used for this technique (which contained 6004 gRNAs, reaching 834 genes) was accurately maintained, thus providing a new platform for studying differences between tissues. In addition, this approach ultimately allows for the evaluation of mutations found in humans and dogs, which is of high relevance for the field [215]. These findings reveal that by using currently available bioengineering tools, 3D in vitro models of primary canine mammary tumors can be generated and used as a preclinical model to investigate mechanisms of carcinogenesis, as well as to screen new therapies for both veterinary and human medicine. Furthermore, both in vitro and in vivo research results must be interpreted together. As in vitro models for breast cancer become more complex, there may be less need for animal-based research in the future.

## 7. Conclusions

Breast cancer is the most frequent neoplasm in women and in intact female dogs worldwide, with uncommon spontaneous occurrence in female rats. To date, there is no effective treatment that can significantly increase the survival rate of breast cancer patients without compromising their quality of life. Despite advances achieved with new diagnostic and therapeutic methodologies, the mortality rate is high in women, and even higher in female dogs, with many owners refusing treatment for economic reasons. Therefore, the use of laboratory animals as models is essential for studying cancer biopathology and evaluating new and more effective therapies, which may improve not only the survival but also the quality of life of oncologic patients. Recently, 3D in vitro tumor models have been developed and have been shown to be promising for studying cancer biopathology and evaluating the performance of candidate therapeutics. These pre-clinical models help to reduce the number of animals used in experimental research and are highly valuable complementary tools. To the best of our knowledge, this is the first review comparing various aspects of the mammary gland and making an integrative analysis of all aspects underlying the development of breast cancer in three different species, ultimately aiming to ensure the consistency and reproducibility of results in future studies. Comparative mammary biology promotes a greater awareness of similarities and differences among species in terms of mammary tumorigenesis, contributing to the acquisition of relevant knowledge about this disease, using a “One Health” approach. The combination of spontaneous and induced models holds potential for finding the answer to breast cancer etiology, and providing novel strategies to screen, prevent and treat this disease in a more effective way. In general, mammary glands in women are more complex than in female dogs and female rats, with more tissue layers and ductal branching. However, the mammary glands in these three species have many similarities that make them useful models for breast cancer research. Nevertheless, it is important to consider the differences when interpreting the results of studies and applying the findings to clinical practice. It is also crucial to understand the anatomical and histological features in order to choose the most appropriate animal model for each line of research.

## Figures and Tables

**Table 1:** Summary of similarities and differences among mammary glands in women, female dogs and female rats.

Feature	Woman	Female Dog	Female Rat
**Number of lactiferous ducts**	10–25 lactiferous ducts	6–16 lactiferous ducts	1 lactiferous duct
**Morphofunctional unit**	Tubuloalveolar	Tubuloalveolar	Tubuloalveolar
**Cells**	Luminal and myoepithelial	Luminal and myoepithelial	Luminal and myoepithelial
**Development of functional glandular unit**	Mature only in pregnancy	Mature only in pregnancy	Mature only in pregnancy
**Vascularization**	Internal thoracic artery Superior thoracic artery Lateral thoracic artery Acromiothoracic artery Thoracodorsal artery Lateral branches of posterior intercostal artery [51,52]	Internal thoracic artery (M1 and M2) Cranial superficial epigastric artery (M3) Caudal superficial epigastric and external pudendal artery (M4 and M5) [15,38]	Superficial cervical, internal thoracic External thoracic and axillary artery (1st and second pairs) Iliolumbar, superficial epigastric and external pudendal arteries (third to sixth pairs) [18]
**Lymphatic drainage**	Axillary lymph nodes and internal mammary lymph nodes (the main) Interpectoral, internal thoracic, supraclavicular, and infraclavicular lymph nodes [48,52]	Axillary lymph nodes (M1, M2 and M3) Superficial inguinal lymph nodes (M3, M4 and M5) [60,61]	Proper axillary node (all pairs) Accessory axillary lymph nodes (1st and second pair) Superficial cervical lymph node (1st pair) Inguinal lymph nodes (fourth, fifth and sixth pairs) [62]
**Nerves**	Third to sixth intercostal nerves second to sixth thoracic intercostal nerves Supraclavicular nerves [52,66]	Fourth, fifth, and sixth thoracic ventral nerves (M1) Sixth and seventh thoracic ventral nerves (M2) Genitofemoral nerve (M3, M4 and M5) [15]	NR

NR—not reported.

## Data Availability

Not applicable.
